# Measuring the data gap: inclusion of sex and gender reporting in diabetes research

**DOI:** 10.1186/s41073-019-0068-4

**Published:** 2019-05-07

**Authors:** Suzanne Day, Wei Wu, Robin Mason, Paula A. Rochon

**Affiliations:** 1 Women’s Xchange, Women’s College Hospital, 76 Grenville Street, Toronto, Ontario M5S 1B2 Canada; 2 Institute for Global Health and Infectious Diseases, University of North Carolina at Chapel Hill, 130 Mason Farm Road, 2nd Floor, Campus Box #7030, Chapel Hill, NC 27599-7030 USA; 3 Women’s College Research Institute, Women’s College Hospital, 76 Grenville Street, Toronto, Ontario M5S 1B2 Canada; 4 Dalla Lana School of Public Health, University of Toronto, 155 College Street, Toronto, Ontario M5T 3M7 Canada; 5 Department of Medicine, University of Toronto, 1 King’s College Circle, Toronto, Ontario M5S 1A8 Canada

**Keywords:** Sex, Gender, Reporting, Research design, Data quality, Diabetes

## Abstract

**Background:**

Important sex and gender differences have been found in research on diabetes complications and treatment. Reporting on whether and how sex and gender impact research findings is crucial for developing tailored diabetes care strategies. To analyze the extent to which this information is available in current diabetes research, we examined original investigations on diabetes for the integration of sex and gender in study reporting.

**Methods:**

We examined original investigations on diabetes published between January 1 and December 31, 2015, in the top five general medicine journals and top five diabetes-specific journals (by 2015 impact factor). Data were extracted on sex and gender integration across seven article sections: title, abstract, introduction, methods, results, discussion, and limitations.

**Results:**

We identified 155 original investigations on diabetes, including 115 randomized controlled trials (RCTs) and 40 observational studies. Sex and gender were rarely incorporated in article titles, abstracts and introductions. Most methods sections did not describe plans for sex/gender analyses; 47 (30.3%) articles described plans to control for sex/gender in the analysis and 12 (7.7%) described plans to stratify results by sex/gender. While most articles (151, 97.4%) reported the sex/gender of study participants, only 10 (6.5%) of all articles reported all study outcomes separately by sex/gender. Discussion of sex-related issues was incorporated into 21 (13.5%) original investigations; however, just 1 (0.6%) discussed gender-related issues. Comparison by journal type (general medicine vs. diabetes specific) yielded only minor differences from the overall integration results. In contrast, RCTs performed more poorly on multiple sex/gender assessment metrics compared to observational studies.

**Conclusions:**

Sex and gender are poorly integrated in current diabetes original investigations, suggesting that substantial improvements in sex and gender data reporting are needed to inform the evidence to support sex- and gender-specific diabetes care.

## Background

Integration of sex (biological factors) and gender (socio-cultural factors) in health research is a crucial step towards improving the quality of evidence to inform and tailor care [1]. There is some evidence to suggest that a sex and gender lens is particularly important in diabetes research. Sex differences have been found across an array of diabetes complications, including microvascular complications, cardiovascular disease, and depression [2]. Sex differences in pharmacological response have also been observed in diabetes treatment [3], with implications for subsequent sex differences in treatment outcomes [4]. For example, males with type 2 diabetes receiving insulin show greater reductions in HbA1c from baseline compared to females, while females are less likely to meet their target HbA1c levels [5]. A qualitative analysis by Mathew et al. [6] found gender differences between men and women in terms of their experiences with diabetes self-management, including differences in disclosure behavior, self-monitoring, and use of diabetes resources [6]. In this study, women were found to more readily disclose their diabetes, better adhere to self-care management strategies, and prefer socially interactive resources for diabetes self-care such as support groups and classes, when compared to men [6].

Evidence pointing to the impact of sex and gender on the experience of and treatment for diabetes has led many to suggest there is a need to develop sex- and gender-specific diabetes care [5–12]. To ensure care providers are equipped with the most complete information to develop such strategies, the potential impact of sex and gender should be routinely explored in the conduct and reporting of diabetes research [3, 10]. Despite increased attention in recent years to sex and gender integration on the part of national funders [13, 14], sex and gender tend to be poorly integrated in medical research publications [15, 16]. Given evidence that diabetes is a disease with clear sex- and gender-specific implications, diabetes research should be integrating consideration of these factors and reporting on the findings as standard practice—for example, by reporting results separately for males and females, discussing differences (and/or similarities) in the resulting outcomes, and including information on what is presently known about diabetes from a sex and/or gender perspective. The extent to which sex and gender are considered and reported on in current diabetes literature is, however, unknown.

We examined full-length, original investigations on diabetes published in 2015 in high-impact general medicine and diabetes-specific journals for reporting on sex and gender considerations. We additionally compared sex and gender reporting by journal type (general medicine vs. diabetes-specific journals) and study type (randomized controlled trials RCTs vs. observational studies). Our findings provide an updated baseline measure of the current state of sex and gender integration in diabetes research.

## Methods

This analysis was conducted on all original investigations on diabetes published between January 1 and December 31, 2015, in ten journals: five general medicine journals and five journals specializing in diabetes research. We selected the top five general medicine journals (by impact factor) listed on the 2015 Journal Citation Reports of ISI Web of Knowledge under the category “Medicine: General and Internal”: *New England Journal of Medicine*, *Lancet*, *Journal of the American Medical Association*, *BMJ*, and *Annals of Internal Medicine*. As there is no category in the Journal Citation Reports for diabetes journals, we selected the top five (by impact factor) diabetes-specific journals under the category “Endocrinology and Metabolism”: *Lancet Diabetes and Endocrinology*; *Diabetes Care*; *Diabetes*; *Diabetologia*; and *Diabetes, Obesity and Metabolism*.

**Fig. 1:**
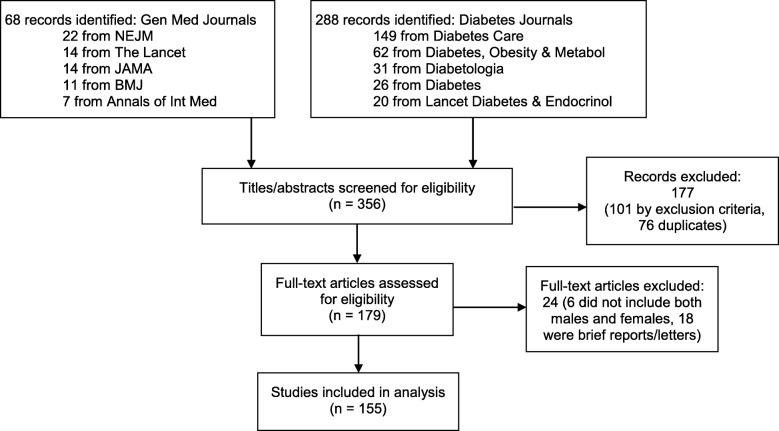
PRISMA flow diagram of search results and study selection. Sixty-eight records of original investigations were initially identified from general medicine (Gen Med) journals, including the *New England Journal of Medicine* (NEJM), *Lancet*, *Journal of the American Medical Association* (JAMA), *BMJ*, and *Annals of Internal Medicine* (Annals of Int Med). Two hundred eighty-eight records were initially identified from diabetes-specific journals, including *Diabetes Care*; *Diabetes, Obesity and Metabolism* (Diabetes, Obesity & Metabol); *Diabetologia*; *Diabetes*; and *Lancet Diabetes and Endocrinology* (Lancet Diabetes & Endocrinol). Titles/abstracts were then screened for eligibility (177 records excluded), followed by full-text assessment of articles (24 articles excluded). A final total of 155 original investigations were included in the analysis

To identify original investigations for inclusion, we searched MEDLINE using “diabetes” in keywords and MESH terms and limiting the search to two types of original investigations: RCTs and observational studies. From these terms, an initial list of 356 original investigations was generated. Inclusion criteria specified that the study had to be designed as an RCT or observational study, and involve a study population composed of both males and females with type 1 or type 2 diabetes. We excluded studies of exclusively pediatric/adolescent study populations, studies that did not include both male and female participants, studies of gestational diabetes, studies that were not RCTs or observational in design, and studies that did not have study populations with type 1 or type 2 diabetes (or that had study populations comprised of both diabetic persons and persons without diabetes or with other conditions). We also excluded research original investigations that were not full-length articles (e.g., research letters or brief reports) as shorter reports may not be expected to adhere to the same level of detail in reporting as a full-length article. Two reviewers (SD and WW) independently reviewed the titles and abstracts of the 356 original investigations (using the criteria above) and compared inclusion/exclusion decisions. Discrepancies were discussed until agreement was reached, producing a final total of 155 original investigations to include in the analysis (see Fig. 1).

A data extraction chart was designed to capture relevant elements of sex and gender reporting in seven sections of each original investigation: title, abstract, introduction, methods, results, discussion, and limitations. This chart was adapted from the Sex and Gender Equity in Research (SAGER) Guidelines [17], a tool to promote comprehensive reporting of sex and gender in published research. Additionally, we documented the type of journal the original investigation was published in (general medicine journal vs. diabetes-specific journal), the type of study conducted (RCT vs. observational study), the percentage of female participants in the study, and whether the terms “sex” and “gender” were used as per the standard definitions used by the National Institutes of Health [18] and the Canadian Institutes of Health Research [14], and the recommended terminology of the SAGER Guidelines [17] and literature on sex- and gender-based health research [19, 20], with sex referring to biological differences between males and females and gender referring to the socio-culturally constructed differences between men, women and gender-diverse persons in terms of roles, behaviors, identities, and distribution of resources. The data extraction chart underwent two additional iterations before being finalized through consensus by the two reviewers (SD and WW) in consultation with a third, arms-length reviewer (RM).

Data were extracted from each of the 155 original investigations. To ensure that no reference to sex/gender was missed, each original investigation was searched electronically using the following terms: sex, gender, male, female, men, and women using either internet browser or PDF-reader search functions. Each section in the data extraction chart contains a set of sex/gender assessment metrics. The combined term “sex/gender” was used in the data extraction chart and for each section was coded as either present (1) or absent (0). It is important to note that our use of the combined term “sex/gender” is not meant to signify that the two terms are interchangeable, but rather as shorthand for “sex and/or gender”, recognizing that for some studies biological sex may be the factor of greatest relevance (e.g., studies of biomedical interventions), while for others the social characteristics associated with gender may be of greater relevance (e.g., studies of health management behaviors).

Additionally, the broadest possible interpretation of sex/gender reporting was used for each assessment metric, encompassing *any mention of sex- and/or gender-related text*. For example, noting the number of females in the study sample in the introduction was coded as present (1) for the assessment metric “sex/gender noted in the introduction”. Similarly, citing gender-related findings from a prior study in the introduction would also be coded as present (1) for the assessment metric “sex/gender noted in the introduction”. We made one exception to the combined assessment of sex/gender: in order to capture nuances in the discussion of sex and gender findings, assessments were made separately for inclusion of sex *and* inclusion of gender in the discussion section of each original investigation. This distinction allowed us to capture whether authors elaborate on their findings in sex- or gender-specific ways. For example, discussing differences or similarities among males and females in terms of glycemic control after medication would be coded as present (1) for the assessment metric “sex issues discussed”, while discussing, for example, potential differences in men’s and women’s medication adherence behaviors would be coded as present (1) for the assessment metric “gender issues discussed”.

Data extraction was conducted in a stepwise manner. The first 50 original investigations were independently analyzed by SD and WW. The reviewers then met to discuss discrepancies in the results of their data extraction. When discrepancies were found, the article was revisited and discussed until consensus was reached. The two reviewers met again after independently reviewing all remaining original investigations to compare coding on each cell in the chart. The initial agreement rate between reviewers was 96.6%, and consensus was reached on all discrepancies with no need for an arbitrator. Results of data extraction were analyzed for all 155 original investigations in total, as well as separately by journal type (articles published in general medicine vs. diabetes-specific journals) and study type (RCTs vs. observation studies).

## Results

We identified 155 original investigations published between January 1 and December 31, 2015, in the ten journals included in our search. Of these 155 original investigations, 23 (14.8%) were published in general medical journals (*New England Journal of Medicine*, *Lancet*, *Journal of the American Medical Association*, *BMJ*, and *Annals of Internal Medicine)* and 132 (85.2%) were published in diabetes-specific journals (*Lancet Diabetes and Endocrinology; Diabetes Care*; *Diabetes; Diabetologia;* and *Diabetes, Obesity and Metabolism*). Of the 155 original investigations, there were 115 (74.2%) RCTs and 40 (25.8%) observational studies. The overall average percentage of female participants in the total 155 original investigations was 41.7%. The results of our assessment of all 155 original investigations, overall and by journal and study type, are presented below in Table 1.

**Table 1:** Assessment of sex/gender integration in original investigations, overall and by journal and study type

		Number of original investigations meeting assessment metric (%)				
Article section	Sex/gender assessment metric	All original investigations *N* = 155	Original investigations in general medicine journals *N* = 23	Original investigations in diabetes-specific journals *N* = 132	Randomized controlled trials *N* = 115	Observational studies*N* = 40
Title & abstract	Sex/gender noted in the title	2 (1.3)	0 (0.0)	2 (1.5)	1 (0.9)	1 (2.5)
	Sex/gender noted in abstract	27 (17.4)	2 (8.7)	25 (18.9)	15 (13.0)	12 (30.0)
Introduction	Sex/gender issues discussed in introduction	15 (9.7)	2 (8.7)	13 (9.8)	5 (4.3)	10 (25.0)
Methods	Plan to control for sex/gender in analysis	47 (30.3)	5 (21.7)	42 (31.8)	23 (20.0)	24 (60.0)
	Plan to stratify results by sex/gender	12 (7.7)	2 (8.7)	10 (7.6)	5 (4.3)	7 (17.5)
Results	Reporting participant sex/gender:					
	• Reported % female participants only	36 (23.2)	6 (26.1)	30 (22.7)	30 (26.1)	6 (15.0)
	• Reported % males participants only	57 (36.8)	9 (39.1)	48 (36.4)	36 (31.3)	21 (52.5)
	• Reported % both male & female participants	58 (37.4)	7 (30.4)	51 (38.6)	45 (39.1)	13 (32.5)
	• Did not report sex/gender distribution of participants	4 (2.6)	1 (4.3)	3 (2.3)	4 (3.5)	0 (0.0)
	Reporting results by sex/gender:					
	• At least one outcome (but not all) reported by sex/gender	24 (15.5)	5 (21.7)	19 (14.4)	17 (14.8)	7 (17.5)
	• All outcomes reported by sex/gender	10 (6.5)	1 (4.3)	9 (6.8)	3 (2.6)	7 (17.5)
	• No outcomes reported by sex/gender	121 (78.1)	17 (73.9)	104 (78.8)	95 (82.6)	26 (65.0)
Discussion	Sex issues discussed	21 (13.5)	0 (0.0)	21 (15.9)	9 (7.8)	12 (30.0)
	Gender issues discussed	1 (0.6)	0 (0.0)	1 (0.8)	1 (0.9)	0 (0.0)
Limitations	Limitations pertaining to sex/gender issues discussed	4 (2.6)	2 (8.7)	2 (1.5)	4 (3.5)	0 (0.0)

Examining the sex/gender assessment results for all original investigations, sex/gender were rarely mentioned in the titles and abstracts of all original investigations reviewed. Sex/gender was mentioned in the introduction section of 9.7% of all original investigations. In descriptions of the study methods, 7.7% of original investigations described plans to examine results separately by sex/gender, and 30.3% of noted that sex/gender would be controlled for in the analysis.

In terms of data reporting, 78.1% of all 155 original investigations did not report any of the findings separately by sex/gender. In 15.5% of original investigations, at least one study outcome (but not all outcomes) was reported separately by sex/gender, and only 10 original investigations in total (6.5%) reported all outcomes separately by sex/gender. Very few original investigations discussed sex issues (13.5%), and only 1 in total (0.6%) included any gender-related discussion. Just 4 original investigations (2.6%) noted potential limitations in relation to sex/gender issues, such as the study cohort consisting mostly of males [21] or females [22], or the need for sex-specific reference limits [23].

The sex/gender of participants was reported in 151 original investigations (97.4%). In terms of sex/gender distribution, 36.8% of all original investigations reported only the proportion of male participants, 23.2% reported only the proportion of female participants, and less than half (37.6%) reported the proportion of both male and female participants. No original investigations provided any indication that data had been collected in a way that was inclusive of sex/gender diversity: only the binary options of male/female (men/women) were present in the original investigations assessed (data not shown).

Comparing the sex/gender assessment of original investigations in general medicine vs. diabetes-specific journals, a slightly greater proportion of original investigations in diabetes-specific journals reported sex/gender in the abstract compared to those in general medicine journals (18.9% vs. 8.7%, respectively). However, a greater proportion of original investigations in diabetes-specific journals did not report any results separately by sex/gender compared to those in general medicine journals (78.8% vs. 73.9%, respectively). Likewise, a smaller proportion of original investigations in diabetes-specific journals discussed limitations pertaining to sex/gender issues compared to those in general medicine journals (1.5% vs. 8.7%, respectively). A slightly greater proportion of original investigations in diabetes-specific journals reported all outcomes by sex/gender compared to those in general medicine journals (6.8% vs. 4.3%, respectively). Finally, the only original investigations discussing sex or gender issues in the discussion section are from diabetes-specific journals.

Comparing the results of sex/gender assessment by study type, a greater proportion of observational studies compared to RCTs noted sex/gender in the abstract (30% vs. 13%, respectively) and introduction (25% vs. 4.3%, respectively). Additionally, more than half (60%) of the observational studies described a plan to control for sex/gender in the methods section, compared to just 20% of RCTs. Although Table 1 shows that very few studies overall report all outcomes separately by sex/gender (6.5%), 17.5% of observational studies did so, compared to just 2.6% of RCTs. While 82.6% of RCTs did not report any data separately by sex/gender, the same is true of 65% of the observational studies. Finally, a greater proportion of observational studies included a discussion of sex issues compared to RCTs (30% vs. 7.8%, respectively).

The results of our assessment of sex/gender terminology use, in all original investigations and by journal and study type, are presented in Table 2.

**Table 2:** Assessment of sex/gender terminology used in original investigations, overall and by journal and study type

Sex/Gender Assessment Metric	All original investigations *N* = 155	Original investigations in general medicine journals *N* = 23	Original investigations in diabetes-specific journals *N* = 132	Randomized clinical trials *N* = 115	Observational studies *N* = 40
Did not use standard terminology for sex/gender	15 (9.7)	1 (4.3)	14 (10.6)	13 (9.7)	2 (5.0)
Did use standard terminology for sex/gender	98 (63.2)	15 (65.2)	83 (62.9)	62 (46.3)	36 (90.0)
Terms sex or gender not used at all	42 (27.1)	7 (30.4)	35 (26.5)	40 (29.9)	2 (5.0)

The majority of all original investigations (63.2%) used standard terminology for sex and/or gender as per accepted definitions [14, 18], for example, using the term sex in descriptions of patient baseline characteristics (typically displayed in the first table of a paper), referring to biological differences between participants. However, 27.1% of original investigations did not use the terms sex or gender at all. In comparing by study type, 10.6% of original investigations published in diabetes-specific journals did not use standard terminology by conflating the terms sex and gender (e.g., using the term gender to refer to biological differences rather than socio-cultural differences), while only 4.3% of those in general medical journals did not use standard terminology. Comparing articles by study type, less than half (46.3%) of RCTs used standard terminology for sex/gender, compared to 90% of observational studies. A far smaller proportion of observational studies declined to use the terms sex or gender at all (5%) compared to RCTs (29.9%).

## Discussion

Our analysis of recently-published, original investigations on diabetes demonstrates low levels of reporting on sex and gender considerations in published diabetes studies overall. While 97.4% of all original investigations reported the sex/gender distribution of the study participants, only 15.5% overall reported at least one outcome by sex/gender, and a mere 6.5% of all reviewed studies reported *all* outcomes of the study separately by sex/gender. In other words, just 6.5% of the original investigations in our review, all of which included both males and females, actually reported all of their study findings a way that would allow the reader to compare results by sex/gender. These studies investigated topics of high importance to the health of persons with diabetes, including cardiovascular risks and complications [24–28], treatment-associated risk of mortality [29, 30], the impact of improvements in fitness and weight [31], racial disparities in risk factor control [32], and the impact of urologic complications on quality of life [33]. Among these studies, the ability to detect differences when comparing results by sex provides important evidence to potentially guide sex-specific diabetes treatment. For example, Belalcazar et al. [31] found novel, sex-specific differences in factors that can modify adiponectin levels (a protein hormone involved in regulating glucose levels): in female patients, improved fitness was shown to be more strongly associated with positive changes in adiponectin levels than weight loss, while in male patients it was weight loss that had the stronger association compared to improved fitness. Conversely, those studies finding no sex differences can help to underscore the relevance of an issue to all persons with diabetes; for example, Zoppini et al. [26] found no statistically significant sex differences in the prevalence of cardiovascular autonomic neuropathy (CAN) among their cohort of patients newly diagnosed with type-2 diabetes. That only 6.5% of our original studies reported all outcomes by sex/gender thus represents a substantial missed opportunity in diabetes literature for investigating the presence or absence of sex/gender differences.

This figure is even lower than that observed in an older study of diabetes management publications between 1975 and 2008, in which 13% of the studies were found to have described sex-/gender-specific differences in the species analyzed in the study and/or conducted sex-/gender-specific analyses [34]. Reviewing the sex-specific features of diabetes more than a decade ago, Legato et al. [7] noted that while some studies in their review included both men and women, a widespread failure to analyze and report on sex differences in these studies outcomes posed a limitation to developing evidence-based recommendations for tailoring care. The baseline measurement of low sex and gender integration observed in our study suggests that this problem unfortunately persists in contemporary publications. Thus, while known sex and gender differences in diabetes complications suggest the value of developing sex- and gender-specific diabetes care [10], our findings on sex/gender reporting indicate that the current literature has a way to go in providing the evidence to develop such tailored strategies.

Although the average percentage of female participants was less than half of all study participants (41.7%), this does represent a step forward from past practices of excluding female participants from scientific research altogether, a history shared by diabetes studies [9]. However, unless study results are reported separately by sex/gender, any additional insights we may have from the inclusion of female participants is lost, limiting a study’s analysis, interpretation, and applicability [15]. The majority of all original investigations in our review (78.1%) did not report any study outcomes by sex/gender, representing a missed opportunity to provide better understanding of differences (or similarities) among study participants.

It is important to acknowledge that it may be difficult for researchers who have not been trained in sex/gender analysis to assess the relevance of sex/gender in their studies, and this may subsequently impact sex/gender reporting practices. While the SAGER Guidelines begin with asking whether sex/gender are relevant to the topic of the study [17], there is no guidance provided for researchers or peer reviewers in order to make this decision. However, prior research has demonstrated substantial evidence that sex and gender are indeed important factors to consider in the field of diabetes research, with implications for diabetes-related complications [2], treatment [3–5], and self-management behaviors [6]. This evidence suggests that conducting sex-/gender-related analyses and reporting on sex/gender data in the context of diabetes research is particularly relevant. For example, knowing whether and how outcomes differ on the basis of sex is an important step in enhancing the effectiveness and safety of diabetes treatments [3], as some interventions may work better for males than females (and vice versa) while adverse events may also differ between males and females. Furthermore, enhancing sex/gender reporting in diabetes research represents an opportunity not only for improving the evidence to tailor diabetes management strategies, but also for identifying interventions that may be of widespread benefit to *all* patients regardless of their sex and/or gender. For example, a recent systematic review has demonstrated that sex does not impact the effectiveness of preventative measures for those with prediabetes [35], pointing to strategies that may benefit both males and females.

The results of our comparisons between journal and study type reveal some additional considerations for enhancing reporting on sex and gender in diabetes research. It is difficult to say which journal type fares better on the sex/gender assessment metrics overall, general medicine vs. diabetes-specific journals. There are, however, greater differences in sex and gender reporting between observational studies and RCTs, with RCTs faring worse on our assessment metrics. Despite being upheld as the “gold standard” of scientific evidence, these findings suggest that the vast majority of RCTs in diabetes research fall far short of providing sufficient evidence from a sex and gender perspective. There is a notable absence of sex and gender criteria in guidelines to improve the quality of RCT reporting, such as the Consolidated Standards of Reporting Trials (CONSORT) Statement [36]. Given their potential implications for research outcomes, we recommend that sex and gender be considered among these standards for excellence in RCT reporting, with standardized criteria for sex and gender reporting such as those offered by the SAGER Guidelines [17]. Additionally, scientific journals can play a role in improving reporting practices by implementing sex and gender reporting policies, such as those adopted as a condition of publication by several high-impact, peer-reviewed general medicine journals [37].

Our study of sex and gender reporting in diabetes literature was limited to original investigations published in 10 journals selected on the basis of impact factor, hence our analysis reflects only a segment of the existing publications in the field of diabetes research. Our use of specific exclusion criteria also limits the generalizability of our findings; it is possible that sex and gender reporting differs in the types of studies that were excluded from our analysis, such as studies of exclusively pediatric populations or studies including persons both with and without diabetes. However, our findings are similar to those of others who have demonstrated sex and gender reporting to be a widespread problem, despite growing recognition of the importance of sex and gender integration on the part of national research funders, including the National Institutes of Health [13] and the Canadian Institutes of Health Research [14]. For example, a review of 60 RCTs published over a 3-month period in 2016 found that just 5% of the studies discussed any sex-related results [16]. Similarly, a review of RCTs published over a 5-year period in high-impact general medicine journals found that data were rarely disaggregated by sex, while the few studies that did disaggregate the data did not discuss any of the sex-/gender-related findings [15]. Reporting on the impact of sex and gender in study findings thus represents one important and under-explored opportunity to improve the yield of research and reduce waste in scientific publications [38], including those on diabetes research.

## Conclusions

The results of our analysis demonstrate that sex and gender data are currently poorly reported in diabetes original investigations in the leading general medicine and diabetes-specific journals. Our findings suggest that improvements in sex and gender data reporting are needed order to provide the best possible evidence to inform tailored diabetes care. To enhance the value of research in the field of diabetes and beyond, future efforts should focus on establishing and enforcing compliance with guidelines for sex and gender reporting requirements, particularly in RCTs.

## Abbreviations

RCT: Randomized controlled trial; SAGER: Sex and Gender Equity in Research
